# Is equitable priority vaccination of vulnerable people feasible in a real-world context? The case of Belgium

**DOI:** 10.1093/eurpub/ckaf075

**Published:** 2025-06-10

**Authors:** Elias Vermeiren, Charlotte Scheerens, Veerle Stouten, John Crombez, Jan De Maeseneer, Joris A F van Loenhout

**Affiliations:** Department of Epidemiology and Public Health, Sciensano, Brussels, Belgium; Department of Public Health and Primary Care, Ghent University, Ghent, Belgium; UNU-CRIS, Bruges, Belgium; Department of Epidemiology and Public Health, Sciensano, Brussels, Belgium; Department of Public Health and Primary Care, Ghent University, Ghent, Belgium; Ghent University Hospital, Ghent, Belgium; Department of Public Health and Primary Care, Ghent University, Ghent, Belgium; WHO Collaborating Center on Family Medicine and Primary Health Care, Ghent University, Ghent, Belgium; Department of Epidemiology and Public Health, Sciensano, Brussels, Belgium

## Abstract

Belgium has implemented a strategy to prioritize vaccination at population level during the COVID-19 pandemic, targeting individuals with pre-existing health conditions at increased risk of severe COVID-19. We aimed to evaluate whether prioritized groups were vaccinated sooner, and which socio-demographic and -economic characteristics were related to the speed of vaccine uptake. We calculated the time to vaccination between the start of the prioritization (1 April 2021) and receiving a first COVID-19 vaccine dose, using this interval as a proxy for evaluating the strategy's early impact. A multivariate regression model, incorporating priority status, age, sex, region of residence, income, and migration background, described the natural logarithm of this time gap. The sample included 4 472 873 individuals vaccinated between 1 April and 31 December 2021, of which 26.4% were prioritized. The results show a 34.6 days earlier vaccination for prioritized individuals versus non-prioritized ones. The time difference between the prioritized and non-prioritized groups was larger in the younger age groups compared to the older age groups (28.2 days versus 19.3 days). Based on the multivariate model estimates, being prioritized [$\beta_{\mathrm{priority}}$ = −0.37, 95% CI (−0.38; −0.36)], older age [$\beta_{55-64}$ = −0.57, 95% CI(−0.58; −0.56)], residency in Brussels or Wallonia [$\beta_{\mathrm{Brussels}}$ = −0.18, 95%CI (−0.20; −0.16); $\beta_{\mathrm{Wallonia}}$ = −0.18, 95% CI (−0.19; −0.17)], having a high income [$\beta_{\mathrm{high}\;\mathrm{income}}$ = −0.11, 95% CI (−0.12; −0.10)], being a Belgian national ($\beta_{\mathrm{belgian}}$ = reference), having had a recent prior infection ($\beta_{\mathrm{no}\;\mathrm{prior}\;\mathrm{infection}}$ = reference) and being female ($\beta_{\mathrm{female}}$ = reference) are associated with a shorter time to vaccination. Developing and implementing a prioritization vaccination strategy accelerated vaccination for the high-risk population with health conditions, demonstrating its feasibility in promoting equitable access to COVID-19 vaccines.

## Introduction

Early 2020 the National Academies of Sciences, Engineering, and Medicine published a report including recommendations and a suggested Framework for Equitable Allocation of COVID-19 vaccines [1], followed by a World Health Organization (WHO) report on equitable access and fair allocation [2]. Belgium proceeded to execute a full-fledged prioritization strategy for individuals with underlying health conditions [3–5].

Decisions on the COVID-19 vaccination strategy in Belgium were taken within the national Task Force Vaccination including prioritization of vaccination [3], although each of the four regional health authorities was responsible for the practical implementation organization. Details on the roll-out of the primary schedule of COVID-19 vaccines among adults in Belgium can be found in Supplementary Material S1. Belgium came to an agreement to prioritize vaccination of the 18–64 years of age with underlying health conditions at higher risk of developing severe COVID-19 (e.g. cardiovascular conditions, respiratory conditions, diabetes mellitus) [6], by descending age, over individuals without underlying health conditions of the same age [7, 8]. It was decided to combine a central selection (using health reimbursement data from the Inter-Mutualistic Agency, IMA) with a decentral selection (using electronic health record data from primary care physicians and a selected group of specialists) based on a combination of databases, since neither of the two data sources contained a complete overview of individuals with underlying health conditions [8]. From 2 April 2021 onwards, the Vaccination Centres could start inviting prioritized citizens by the central IMA-selection. The decentralized selection was uploaded from 13 April 2021 onwards [8]. The vaccine invitation system was uniform for all Belgian residents, independent of priority status or selection method among prioritized individuals: persons were proposed an appointment to get vaccinated or were able to make an appointment at the vaccination centre to which they were assigned and were allocated a vaccination code to obtain their vaccine [8]. Every person had the possibility to check whether they were prioritized online [9].

Besides prioritization strategies in vaccination campaigns, also individuals’ characteristics may influence the speed of vaccine uptake. Several studies have shown the impact of belonging to certain socio-economic and -demographic groups and having had a prior infection on COVID-19 vaccine uptake [10–15]. A study in Belgium showed that individuals with a lower education, those belonging to a lower income group, and those with a migration background had a relatively low uptake of the first dose of a COVID-19 vaccine [14].

The aim of this study was to assess the prioritization strategy that identified individuals with underlying health conditions at a heightened risk of severe COVID-19. We aimed to determine the extent to which this strategy facilitated an equitable distribution of COVID-19 vaccines by comparing the vaccination uptake speed between prioritized individuals with underlying health conditions and those without, within the Belgian context. Additionally, the impact of the prioritization strategy across various demographic and socio-economic variables was explored within this study.

## Methods

### Study population

We focused on inhabitants of Belgium who were alive on 1 April 2021 and fell within the age range of 18–64 years at the time of vaccination. Our analysis included individuals who received at least one dose of the COVID-19 vaccine between 1 April and 31 December 2021. The study population was divided into two groups: Group 1 comprised individuals selected for vaccination prioritization due to having underlying health conditions. Group 2 consisted of individuals who were vaccinated according to the standard procedure, without immediate underlying health conditions. Given that healthcare workers were prioritized in the first phase of the vaccination campaign before individuals with underlying health conditions, individuals with a healthcare licence (serving as a proxy for healthcare workers) were excluded.

### Data sources

Sciensano, the Belgian Institute for Public Health, set up a surveillance of COVID-19 vaccination, called the LINK-VACC project: selected variables from seven existing national health and social sector registers were linked at an individual level using the national registry number within a secured pseudonymized environment. Within this study, we did not use variables from the COVID-19 clinical hospital survey, as it consists of a non-exhaustive surveillance of patients hospitalized for COVID-19, and as such it was not relevant for the objectives of this study. Potentially relevant variables from each of the used datasets were preselected, with the justification that they might impact the speed of vaccine uptake in Belgium. This selection was based on findings of related studies [10–14]. For the present study, the following databases were used:

1. The Vaccination Codes DataBase (VCDB) which is the database on a federal level containing the identification of selection for prioritization, whether an individual was invited with priority.
2. VaccinNet+, the COVID-19 vaccine registry for all Belgian residents, containing demographical data of the vaccinated person (sex, year of birth, postal code of residence) and data on the administered vaccine (e.g. date of administration).
3. The Statbel DEMOBEL database provided by the Statistics Belgium: demographical data (such as migration background, household status) and socio-economic data (such as level of education, employment status, income level) of all Belgian citizens who have received at least one COVID-19 vaccine and/or have been tested for COVID-19 in Belgium.
4. Database of the Inter-mutualistic Agency (IMA): data on morbidity, indirectly defined through reimbursement of medication for conditions with a higher risk of developing severe COVID-19 of all persons affiliated to a Belgian health insurance fund, and who have received at least one COVID-19 vaccine and/or have been tested for COVID-19 in Belgium.
5. The Common Base Registry for HealthCare Actors (CoBRHA), containing information on individuals who have been licenced to practice a healthcare profession in Belgium (by profession and specialty).
6. Laboratory Test database, containing information on individuals who have been tested for COVID-19, and the outcome of this test (positive or negative)

### Variables and measures

The administration of the first vaccine dose in Belgium among the prioritized population (based on underlying health conditions) started after 1 April 2021. We defined the initial explanatory variable as the priority status and the outcome variable, as the Gap, namely the time difference in days between 1 April 2021 and the actual vaccination date of a first COVID-19 vaccine dose. Since the individual vaccine invitation date was not available for this study, we could not measure the day difference between the vaccine invitation date and vaccine administration date. Hence, the main hypothesis tested is the time difference in vaccine uptake between the prioritized group and the non-prioritized group after start of the prioritization strategy.

Additionally, we identified the effect of other characteristics on the Gap in a multivariate model, for which we used a set of socio-demographic and -economic variables: age (at vaccine administration, categorized into five groups: 18–24, 25–34, 35–44, 45–54, and 55–64 years); sex (female or male as registered in the national registry); region (Flanders, Wallonia, Brussels, based on the postal code in the national registry); education [classified in eight categories using the International Standard Classification of Education (ISCED) and merged into three main education levels: low (ISCED0–ISCED2), moderate (ISCED3–ISCED4), and high (ISCED5–ISCED8)]; income [categorized into low income (deciles 1–3), moderate income (deciles 4–7), and high income (deciles 8–10)]; employment (employed, unemployed, or unknown, as registered by social security); migration background [which is based on nationality, categorized in domestic (Belgian nationality with Belgian parents), foreign (no Belgian nationality), or foreign parent (at least one parent with no Belgian nationality)]; immunocompromised (yes/no, dependent on whether a person had received immunosuppressants or medication for treating an immunocompromising condition) and having had a prior COVID-19 infection (yes/no) between 1 January 2021 and the first vaccine dose administration. Only individuals for whom all considered variables were complete were selected for our analyses.

### Statistical analysis

The outcome variable, Gap, is lognormally distributed in the dataset. We have applied a multivariate linear model with the natural logarithm of Gap as outcome and priority status, age, sex, region, income, and migration background as fixed variables. Estimates and 95% confidence intervals were obtained by bootstrapping. The linear model was applied a 5000-fold to a 10% random sample of the cleaned data. Standard deviations were scaled and the median was used as estimate per level. Model coefficients were exponentiated to estimate effect size.

The following formula was used for the multivariate linear model:


$$\ln\left(\mathrm{Gap}\right)=\beta_{0}+\sum_{i=1}^{n}\beta_{i}X_{i}$$

In which $\beta_{0}$ represents the reference group, i.e. non-prioritized; age between 18 and 24 years old; female sex; Flanders as region of residence; low income and domestic; not having had a prior COVID-19 infection between 1 January 2021 and first vaccine administration. n represents the number of levels across all variables without reference levels. Unpaired Welch’s *t*-tests were used to assess whether the weighted means of the natural logarithm of Gap significantly differed between groups.

Fixed variables were selected by multicollinearity assessment [generalized variance inflation factor (GVIF)] combined with stepwise regression. In the latter, the model with the smallest number of fixed variables explaining the most variance or lowest Akaike’s information criterion (AIC) was chosen. Education and employment were omitted due to correlation with income (GVIF ≥ 2) and higher AIC (AIC_income_ = 3.59 × 0^6^, AIC_education_ = 3.60 × 10^6^, AIC_employment_ = 3.66 × 10^6^). The source year for the income variable is also more recent compared to the education variable (2021 versus 2017). ‘Immunocompromised’ was also excluded because of the conceptual overlap with the main explanatory variable of priority status and higher univariate AIC. All executed tests were significant. Bootstrapping was applied to verify robustness of the coefficients and confidence intervals in the multivariate model. All statistical analyses and visualizations were performed in R version 4.1.3.

### Ethics approval

The study was conducted according to the guidelines of the Declaration of Helsinki. The protocol of the LINK-VACC project was approved by the medical ethics committee University Hospital Brussels—on 3 February 2021 (reference number 2020/523) and obtained authorization from the Information Security Committee (ISC) Social Security and Health (reference number IVC/KSZG/21/034). Informed consent was waived based on Article 6 §1(e) and Article 9 §2(i) of the General Data Protection Regulation (see ethical statement).

## Results

### Characteristics of the study sample

Out of 4 811 462 vaccinated individuals, aged 18–64 years, 4 472 873 individuals had complete information on demographic and socio-economic status and were included for this study (Table 1, Supplementary Material S2), meaning that the final sample included in the analyses represented 93% of the study population. After checking missingness among all study variables (stratified by priority status), none of them had more than 6% of missing values. In this study sample, 1 126 310 (26.4%) belonged to the priority group and 3 290 563 (73.6%) to the non-priority group. Included persons had an average age of 42.1 ± 13.3 years, the largest categories were those who had a medium level of education (33.6%), those who were male (53.9%), and had a medium income (38.6%). Individuals invited in priority were on average older (52.6 ± 9.48 versus 38.3 ± 12.5 years) and 51.9% of them was between 55 and 64 years old (versus 12.9% among non-prioritized). Prioritized and non-prioritized individuals closely resembled each other in terms of other characteristics, except that those prioritized were slightly more often Belgian nationals (75.8% versus 69.1%), and living in Flanders (64.5% versus 62.0%).

**Table 1.** Sample characteristics

		Group 1: priority		Group 2: non-priority		
Variable	Level	*N*	(%)	*N*	(%)	Total
Full sample		1 182 310	26.4^b^	3 290 563	73.6^b^	4 472 873
Age	18–24	23 924	2.0^a^	556 940	16.9^a^	580 864
	25–34	54 774	4.6^a^	814 239	24.8^a^	868 103
	35–44	97 891	8.3^a^	846 198	25.7^a^	944 089
	45–54	393 365	33.3^a^	648 778	19.7^a^	1 042 143
	55–64	613 356	51.9^a^	424 318	12.9^a^	1 037 674
Biological sex	Female	549 464	46.5^a^	1 513 848	46.0^a^	2 063 312
	Male	632 846	53.5^a^	1 776 715	54.0^a^	2 409 561
Region	Flanders	762 484	64.5^a^	2 041 309	62.0^a^	2 803 793
	Brussels	77 749	7.6^a^	276 257	8.4^a^	354 006
	Wallonia	342 077	28.9^a^	972 997	29.6^a^	1 315 074
Income level	Low	270 472	22.9^a^	724 924	22.0^a^	995 396
	Medium	455 174	38.5^a^	1 271 917	38.7^a^	1 727 091
	High	449 664	38.0^a^	1 225 723	37.2^a^	1 675 387
	Unknown	7000	0.6^a^	67 999	2.1^a^	74 999
Migration background	Belgian national	895 900	75.8^a^	2 272 373	69.0^a^	3 168 273
	Foreign	255 907	21.6^a^	765 232	23.3^a^	1 021 139
	Foreign parent	30 503	2.6^a^	252 958	7.7^a^	283 461
Prior infection status	No prior infection	1 141 440	96.5^a^	3 147 117	95.6^a^	4 288 557
	Prior infection	40 870	3.5^a^	143 446	4.4^a^	184 316

Sample characteristics per priority groups across all variables used in the multivariate model. The first row represents the full sample and its proportions per priority group. All other rows represent the number of individuals per respective level and priority group, percentages are variable proportions within each priority group. The ‘Total’ column depicts the total number of individuals per level.

a Column percentages, representing the proportion of each level per respective variable.

b Row percentages, representing the proportion of each priority group over total sample size.

### Assessment of speed of vaccination uptake between (non-)prioritized

**Figure 1.**
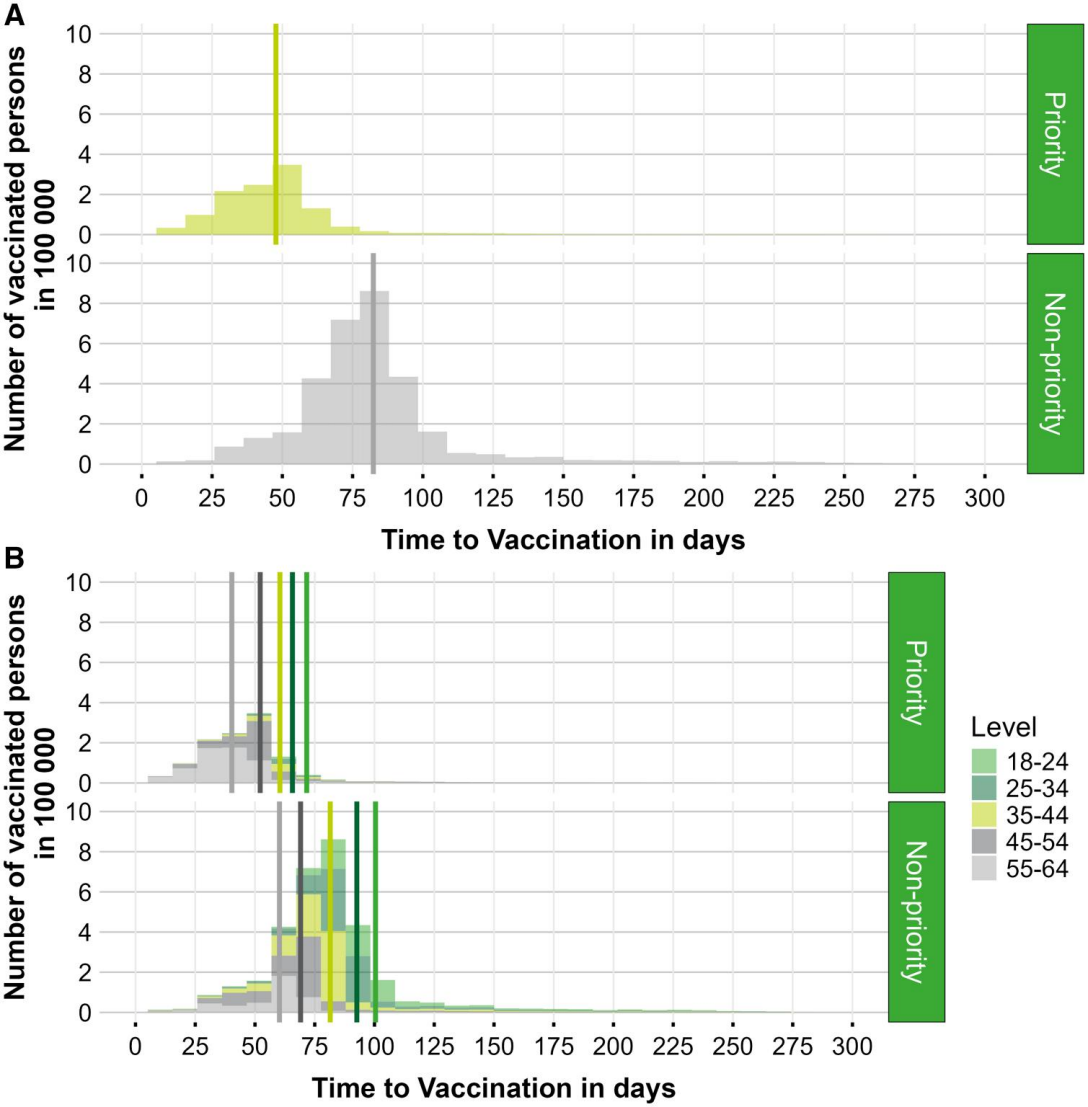
‘Time to vaccination’ is the day difference between 1 April 2021 and the date of COVID-19 first vaccine dose administration. (A) The coloured vertical lines depict the mean time to vaccination per priority group. Priority: 47.7 days; non-priority: 82.3 days. (B) The coloured vertical lines depict the mean time to vaccination in days per age category and priority group. From left to right: 55–64 priority: 40.3 days; 55–64 non-priority: 60.2 days; 45–54 priority: 52.2 days; 45–54 non-priority: 69.1 days; 35–44 priority: 60.4 days; 35–44 non-priority: 81.5 days; 25–34 priority: 65.6 days; 25–34 non-priority: 92.6 days; 18–24 priority: 71.7 days; 18–24 non-priority: 101.0 days.

Using histograms (Fig. 1A), we present the univariate analysis of the Gap variable, which represents the number of days between 1 April 2021 and the date of administration of the first dose of a COVID-19 vaccine. The mean Gap for the prioritized group was 47.7 days (±26.5), compared to 82.3 days (±32.8) for the non-prioritized group, showing a statistically significant difference with a mean Gap reduction of 34.6 days (82.3 minus 47.7) favouring the prioritized group (*t* = 1141.3, df = 2 558 601). Over 90% of individuals in the prioritized group received their first vaccine dose before the average Gap observed in the non-prioritized group.

To disentangle the effect from the prioritization strategy and the overall vaccination strategy based on descending age, we stratified the same analysis by age (Fig. 1B). Across all age cohorts within the priority group, vaccinations were administered earlier than in their non-priority counterparts. The smallest difference was observed in the 45–54 age cohort, at 16.9 days (69.1 days for the priority group minus 52.2 days for the non-priority group). The largest difference was noted in the 18–24 age cohort, at 29.3 days. For the 55–64, 35–44, and 25–34 age cohorts, the differences in mean gap were 19.9, 21.1, and 27.0 days, respectively.

### Multivariate estimates

Table 2 displays the outcomes derived from the multivariate model, indicating the relative estimates of selected variables compared to their reference groups. All estimates are highly significant (*P* values <.001). Individuals invited with priority were associated with a significant reduction in the time to vaccination compared to the non-prioritized ($\Delta_{\mathrm{priority}}$ = 32.6 days for the reference population, $\beta_{\mathrm{priority}}$ = −0.37, 95% CI (−0.38; −0.36)). In comparison with the youngest age group, we observed the largest day difference in the oldest age group and note an increasing $\Delta_{\mathrm{days}}$ and decreasing estimate along increasing age. Individuals living in Brussels and Wallonia were on average 17.4 days earlier vaccinated compared to their peers living in Flanders. Persons with a medium or high income received their first COVID-19 vaccine dose faster compared to persons with a low income. We observed a slower vaccine uptake among men compared to women, in individuals with a migrant background compared to domestic individuals, and in individuals who had a prior COVID-19 infection from 1 January 2021 onwards compared to those without a prior infection in this period.

**Table 2.** Multivariate model coefficients

Variable	Level	$\beta_{\mathrm{adj}}$	95% CI	$T_{\mathrm{vaccination}}$ ^a^ (days)	95% CI	Δ ^b^ (days)
Intercept		4.66	(4.65; 4.67)	105.6	(103.6; 106.1)	0
Priority (ref.: non-priority)	Priority	−0.37	(−0.38; −0.36)	73.0	(70.9; 74.7)	32.6
Age (ref.: 18–24)	25–34	−0.08	(−0.09; −0.07)	97.5	(94.7; 100.0)	8.1
	35–44	−0.21	(−0.22; −0.20)	85.6	(83.2; 87.8)	20.0
	45–54	−0.36	(−0.37; −0.35)	73.7	(71.6; 75.6)	31.9
	55–64	−0.57	(−0.58; −0.56)	59.7	(58.0; 61.3)	45.9
Biological sex (ref.: female)	Male	0.01	(0.01; 0.02)	106.7	(103.9; 109.1)	−1.1
Region (ref.: Flanders)	Brussels	−0.18	(−0.20; −0.16)	88.2	(85.2; 91.0)	17.4
	Wallonia	−0.18	(−0.19; −0.17)	88.2	(85.8; 90.3)	17.4
Income (ref.: low income)	High income	−0.11	(−0.12; −0.10)	94.6	(92.0; 97.0)	10.0
	Medium income	−0.07	(−0.08; −0.06)	98.5	(95.7; 100.9)	7.1
	Unknown	−0.02	(−0.04; 0.01)	103.5	(99.2; 107.6)	2.1
Migration background (ref.: domestic)	Foreign	0.09	(0.08; 0.10)	115.6	(112.3; 118.4)	−10.0
	Foreign parent	0.07	(0.06; 0.09)	113.3	(109.7; 116.5)	−7.7
Prior infection status^c^ (ref.: no prior infection)	Prior infection	0.13	(0.11; 0.15)	120.3	(116.1; 124.1)	−14.7

Multivariate model estimates per level. $\beta_{\mathrm{adj}}$ represent adjusted median model estimates obtained by bootstrapping 10% of the data 5000-fold. Confidence intervals were obtained by applying scaling to the standard deviation. $T_{\mathrm{vaccination}}$ represents the average time to vaccination in days relative to 1 April 2021 for the row level keeping other levels as their reference. Δ represents the difference between $T_{\mathrm{intercept}}$ and $T_{\mathrm{level}}$ in days.

Adjusted multivariate estimates with 95% confidence intervals per fixed variable.

Abbreviations: CI: confidence interval, ref.: reference level.

a Time to vaccination in days relative to 1 April 2021. Calculated as exp(β_intercept_ + β_row_).

b Day difference between intercept and row coefficient. Calculated as exp(β_intercept_) − exp(β_intercept_ + β_row_).

c Prior infection status between 1 January 2021 and date of vaccine administration.

The results in Table 2 can be used to calculate the average time to vaccination for a specific sub-group, e.g. for non-prioritized men, aged between 55 and 64, living in Flanders, low income, foreign, and prior infection. This can be calculated as follows: $\exp\left(\beta_{0}+\beta_{55-64}+\beta_{\mathrm{male}}+\beta_{\mathrm{foreign}}+\beta_{\mathrm{infection}}\right)=\exp\left(4.66-0.57+0.01+0.09+0.13\right)=75.2\;$ days. If we calculate the average time to vaccination for the same group but prioritized, the following is obtained: $\exp\left(\beta_{0}+\beta_{\mathrm{priority}}+\beta_{55-64}+\beta_{\mathrm{male}}+\beta_{\mathrm{foreign}}+\beta_{infection}\right)=\exp\left(4.66-0.37-0.57+0.01+0.09+0.13\right)=51.9$ days. The Δ between these two groups, equals 23.3 days.

## Discussion

### Summary of main findings

This study assessed for Belgium its comprehensive (central and decentral), proactive, and equitable prioritization vaccination strategy at population level during the COVID-19 pandemic. The strategy targeted individuals with pre-existing health conditions, who were at increased risk of severe COVID-19. The unadjusted day difference is 34.6 days between the prioritized and non-prioritized, in favour of the prioritized. Additionally, it was shown that socio-economic and socio-demographic factors also impacted the speed of vaccine uptake, although to a lesser extent than the prioritization strategy. The variable ‘age’ had a larger impact, as expected due to the strategy that was used. Finally, having had a recent prior COVID-19 infection between 1 January 2021 and the date of vaccine administration reduced the speed of vaccine uptake as well.

### Interpretation of results

The observed average earlier vaccination of the prioritized group by 34.6 days compared to the non-prioritized group was partially attributed to age. Prioritized individuals were on average older and half of them was between 55 and 64 years old (versus 12.9% among non-prioritized). Our stratified bivariate analysis showed that within each age group prioritized individuals were vaccinated earlier than their non-prioritized counterparts, but with a varying magnitude of the mean Gap difference by age group, being smaller for older age groups. Our results from the multivariate analysis indicated that mainly older age groups were inversely associated with the speed of vaccine uptake (β = −0.57, 95% CI −0.58 to −0.56 for 55–64-year-olds) as was expected because of the impact of the overall vaccination strategy by descending age. However, when taking age groups and other confounders into account, being prioritized was still associated with a significant reduction in the time to vaccination (β = −0.37; 95% CI −0.38 to −0.36; difference to intercept in days of 32.6 days for the reference population). Therefore, the impact of the prioritization strategy was only partially attributable to having an older age, indicating a nuanced impact of prioritization strategies across different demographic segments. This was due to limited vaccination capacity at that time [4, 5], and a relatively late vaccination of people in younger age groups who were not prioritized.

The advancement of vaccination by the prioritization strategy by approximately one month may seem quite marginal. However, the extent of advancement in vaccination schedules is inherently contingent upon the vaccine's availability within a particular country and context and the efficiency of its distribution networks. As analysed by the European Centre for Disease Prevention and Control (ECDC) [16], Belgium was on fifth place among all EU/EEA countries in terms of the cumulative uptake of the primary scheme of COVID-19-vaccination by week 37 in 2021. In scenarios characterized by constraints in vaccine production or in countries with less efficient distribution mechanisms or who faced more substantial challenges, the effect of a prioritization strategy might be considerably more pronounced.

Adjusting for a broader set of variables also revealed that vaccine uptake was significantly slower among individuals with a migration background and those with a lower income (or educational attainment, education was collinear to income). A previous study in Belgium showed that overall COVID-19 vaccine uptake among these groups was also lower compared to individuals without a migration background and those with higher or medium income, respectively [14]. Various international studies show similar results in vaccine uptake related to migration background or ethnicity and socio-economic factors [17–19]. Our findings are important to consider and to guide implementations in future vaccination campaigns (e.g. targeted communication in different languages, and through specific media). Persons with a recent prior COVID-19 infection had a slower uptake than those without a recent infection. This is possibly due to the fact that recent prior infections confer a high level of protection against a symptomatic infection, as confirmed in a previous study [20], which might have been assumed and served as an argument for individuals to get vaccinated later.

We also observed a difference in the uptake speed between the three Belgian regions in the multivariate model, with a faster uptake in Brussels and Wallonia compared to Flanders. In previous reports, however, the overall vaccine uptake was shown to be lower in Brussels and Wallonia [14, 21]: the coverage for primary series in adults was 92% in Flanders, 81% in Wallonia, and 68% in Brussels by 31 October 2021 [21]. As such, when overall uptake was relatively low in a given region, it allowed this region to proceed faster with inviting the next individuals on the waiting list, increasing the speed of vaccine uptake. Interestingly the differences in coverage rates resonate with the percentage of people that have a General Medical Record with a General Practitioner, centralizing all patient’s medical information: respectively 82% in Flanders, 69% in Wallonia, and 59% in Brussels [8]. As a reflection we could hypothesize that these associations could potentially be explained by the relation and trust between patients and their health care providers (in this case the GPs) and the health system organization (vaccination centres organized in primary care) [22]. This hypothesis could be subject of future field testing.

### Strengths and limitations

Our results give a unique first insight into the applicability and implementation of prioritizing people with underlying health conditions in a real-world setting, which could inform future vaccination campaigns. We used an extensive study sample, 4.5 million vaccinated individuals of which 1.2 million invited with priority, the latter representing most of the entire population of interest of 1.5 million persons invited with priority in Belgium.

There are also a few limitations that may have impacted our study. We did have an extensive sample of individuals invited with priority in our linked registry, but not the exhaustive full population. Consequently, we were unable to calculate the vaccine coverage among both prioritized and non-prioritized groups. Additionally, we did not have the exact date on which everyone was invited for vaccination, so we could only assess whether the prioritized group was vaccinated faster than the non-prioritized group in relation to the start of the prioritization strategy on 1 April 2021. Our final study sample was reduced by 7% due to missing values in our study variables, and our decision to only include individuals with complete data. This reduction is likely a consequence of automated data linkage of the large datasets, and we do not expect this to impact our results. Finally, we only assessed the impact of observational descriptive variables on vaccine uptake and did not have information available on other potentially influential factors such as religion, and trust in vaccine efficacy and safety, which are shown to influence vaccination willingness [23, 24].

## Conclusions

In this study, we evaluated the feasibility of a priority vaccination strategy based on underlying health conditions during the COVID-19 pandemic, focusing on the Belgian population aged 18–64 years. Our findings demonstrate that this strategy was successful in ensuring that vulnerable individuals were vaccinated, on average, slightly more than one month earlier than those not prioritized. Our analysis also reveals significant disparities in speed of vaccination uptake related to income, education level, and migration background. These disparities highlight critical challenges that must be addressed in the future development and implementation of vaccination prioritization strategies to ensure equitable access and uptake across all segments of the population.

## Supplementary Material

ckaf075_Supplementary_Data

## Data Availability

As the LINK-VACC project is not an open-access platform, the individual level data are only available to researchers working on the project. However, general descriptive statistics from the registries used in LINK-VACC are available from https://epidata.sciensano.be/epistat/dashboard/#covid. This includes inter alia the number of confirmed cases by date, age, sex, and province or the number of administered vaccines by date, region, age, sex, brand, and dose.
