# Supplementary material for: Is equitable priority vaccination of vulnerable people feasible in a real-world context? The case of Belgium
Source: Eur J Public Health. 2025 Jun 10;35(4):774–80. doi: 10.1093/eurpub/ckaf075 (PMC12312844; doi:10.1093/eurpub/ckaf075)
Supplement: ckaf075_Supplementary_Data [file ckaf075_supplementary_data.docx]

## **Table S1. Target groups for the primary schedule of COVID-19 vaccines by month**

| **Month** | **Who** |
| --- | --- |
| January 2021 | Residents and staff in nursing homes + nursing staff (doctors, nurses, etc.) in hospitals |
| February 2021 | Primary care staff (general practitioners, pharmacists, etc.) + collective care institutions (disability care, etc.) + other hospital staff |
| March - April 2021 | Everyone aged 65 years and over (from oldest to youngest) |
| April - May 2021 | People aged 18 to 64 years at higher risk due to underlying health conditions (from oldest to youngest) |
| June 2021 | Everyone aged 18 years and over (from oldest to youngest) |

*Translated and adapted from Catteau et al., 2021.^[[1]](#footnote-1)^*

## **Table S2. Additional sample characteristics.**

|  |  | Group 1: Priority | | Group 2: Non-priority | |  |  |
| --- | --- | --- | --- | --- | --- | --- | --- |
| Variable | **Level** | **N** | **(%)** | **N** | **(%)** | **Total** |  |
| Education | Unknown | 107 331 | 9.1 | 383 735 | 11.7 | 500 924 |  |
|  | Low education | 366 981 | 31.0 | 860 941 | 26.2 | 1 246 432 |  |
|  | Medium education | 419 390 | 35.5 | 1 084 292 | 33.0 | 1 515 037 |  |
|  | High education | 288 608 | 24.4 | 961 595 | 29.2 | 1 255 409 |  |
| Immunocompromised | Not immunocompromised | 1 128 529 | 95.5 | 3 276 399 | 99.6 | 4 449 604 |  |
|  | Immunocompromised | 53 781 | 4.5 | 14 146 | 0.4 | 68 198 |  |
| Province | Brussels Capital Region | 77 749 | 6.6 | 276 257 | 8.4 | 354 006 |  |
|  | Antwerpen | 206 762 | 17.5 | 582 391 | 17.7 | 789 153 |  |
|  | Hainaut | 133 868 | 11.3 | 355 125 | 10.8 | 488 993 |  |
|  | Limburg | 112 571 | 9.5 | 254 968 | 7.7 | 367 539 |  |
|  | Liège | 103 266 | 8.7 | 285 400 | 8.7 | 388 666 |  |
|  | Luxembourg | 21 215 | 1.8 | 63 512 | 1.9 | 84 727 |  |
|  | Namur | 47 873 | 4.0 | 146 465 | 4.5 | 194 338 |  |
|  | Oost-Vlaanderen | 179 813 | 15.2 | 494 682 | 15.0 | 674 504 |  |
|  | Vlaams-Brabant | 122 519 | 10.4 | 347 258 | 10.6 | 469 777 |  |
|  | Brabant-Wallon | 35 855 | 3.0 | 122 495 | 3.7 | 158 350 |  |
|  | West-Vlaanderen | 140 820 | 11.9 | 362 000 | 11.0 | 502 820 |  |
| Source (Reason for prioritisation) | None | 568 | 0.0 | 3 291 131 | 100.0 | 3 291 131 |  |
|  | NIC | 513 584 | 43.4 | NA | NA | 513 584 |  |
|  | NIC, GP | 364 665 | 30.8 | NA | NA | 364 665 |  |
|  | GP | 302 698 | 25.6 | NA | NA | 302 698 |  |
|  | NIC, Hospital | 297 | 0.0 | NA | NA | 297 |  |
|  | NIC, GP, Hospital | 116 | 0.0 | NA | NA | 116 |  |
|  | GP, Hospital | 160 | 0.0 | NA | NA | 160 |  |
|  | Hospital | 222 | 0.0 | NA | NA | 222 |  |
| Employment | Employed | 877 152 | 74.2 | 2 488 563 | 75.6 | 3 365 715 |  |
|  | Unemployed | 301 884 | 25.5 | 760 420 | 23.1 | 1 062 304 |  |
|  | Unknown | 3274 | 0.3 | 41 58 | 1.3 | 44 854 |  |
| Abbreviations: NIC: national inter-mutualistic college; GP: general practitioner; NA: Not available | | | | | | | |

Legend: Sample characteristics per priority groups across all variables that were not used in the multivariate model. All rows represent the number of individuals per respective level and priority group, percentages are variable proportions within each priority group. The ‘Total’ column depicts the total number of individuals per level.

1. Catteau L., van Loenhout J., Stouten V., Billuart M., Hubin P., Haarhuis F., Wyndham Thomas C. Couverture vaccinale et impact épidémiologique de la campagne de vaccination COVID-19 en Belgique. Données jusqu’au 31 octobre 2021 inclus. Bruxelles, Belgique: Sciensano ; 2021. Numéro de dépôt légal : D/2021/14.440/79
   <https://covid-19.sciensano.be/sites/default/files/Covid19/COVID-19_THEMATIC_REPORT_VaccineCoverageAndImpactReport_FR.pdf> [↑](#footnote-ref-1)
